# Gene therapy with RALA/iNOS composite nanoparticles significantly enhances survival in a model of metastatic prostate cancer

**DOI:** 10.1186/s12645-018-0040-x

**Published:** 2018-06-01

**Authors:** Cian M. McCrudden, John W. McBride, Joanne McCaffrey, Emma M. McErlean, Nicholas J. Dunne, Vicky L. Kett, Jonathan A. Coulter, Tracy Robson, Helen O. McCarthy

**Affiliations:** 1 School of Pharmacy, Queen’s University Belfast, 97 Lisburn Road, Belfast, BT9 7BL Northern Ireland, UK; 2 School of Mechanical and Manufacturing Engineering, Dublin City University, Dublin, Ireland; 3 Molecular and Cellular Therapeutics, Royal College of Surgeons in Ireland, 123 St Stephen’s Green, Dublin 2, Ireland

**Keywords:** Prostate cancer, Nitric oxide, Non-viral gene therapy, Amphipathic peptide, iNOS

## Abstract

**Background:**

Recent approvals of gene therapies by the FDA and the EMA for treatment of inherited disorders have further opened the door for assessment of nucleic acid pharmaceuticals for clinical usage. Arising from the presence of damaged or inappropriate DNA, cancer is a condition particularly suitable for genetic intervention. The RALA peptide has been shown to be a potent non-viral delivery platform for nucleic acids. This study examines the use of RALA to deliver a plasmid encoding inducible nitric oxide synthase (iNOS) as an anti-cancer treatment.

**Methods:**

The physiochemical properties of the RALA/DNA nanoparticles were characterized via dynamic light scattering and transmission electron microscopy. The nanoparticles were labelled with fluorophores and tracked over time using confocal microscopy with orthogonal sections to determine cellular location. In vitro studies were employed to determine functionality of the nanoparticles both for pEGFP-N1 and CMV-iNOS. Nanoparticles were injected intravenously into C57/BL6 mice with blood and serum samples analysed for immune response. PC3-luc2M cells were injected into the left ventricle of SCID mice followed by treatment with RALA/CMV-iNOS nanoparticles to evaluate the tumour response in a metastatic model of prostate cancer.

**Results:**

Functional cationic nanoparticles were produced with gene expression in PC-3 prostate cancer cells. Furthermore, repeated administrations of RALA/DNA nanoparticles into immunocompetent mice did not produce any immunological response: neutralization of the vector or release of inflammatory mediators. RALA/CMV-iNOS reduced the clonogenicity of PC-3 cells in vitro, and in an in vivo model of prostate cancer metastasis, systemically delivered RALA/CMV-iNOS significantly improved the survival of mice.

**Conclusion:**

These studies further validate RALA as a genetic cargo delivery vehicle and iNOS as a potent therapy for the treatment of cancer.

## Background

Despite recent advances in gene therapy, including the approval of Glybera^®^ for treatment of lipoprotein lipase deficiency, the safety, cost and patient perception issues that have dogged viral strategies for gene therapy persist. This has created opportunities for the development of non-viral technologies such as cationic polymers, lipoplexes, or peptides for nucleic acid delivery (Pahle and Walther 2016). RALA is an example of a peptide delivery vehicle (McCarthy et al. 2014). RALA is a 30 amino acid amphipathic fusogenic peptide (N-WEARLARALARALARHLARALARALRACEA-C) with seven arginines in the backbone that facilitate condensation of nucleic acids. When RALA is incubated with anionic entities, electrostatic interactions facilitate the spontaneous production of nanoparticles. The RALA system is highly tunable with variations in the molar ratios of peptide and DNA (the *N*:*P* ratio) modulating size and charge characteristics (McCarthy et al. 2014). The RALA nanoparticles readily traverse cell membranes, owing to not only the arginines, but also the six leucines, all of which facilitate interaction between the nanoparticle and the cell membrane. RALA/DNA nanoparticles penetrate ZR-75-1 and NCTC-929 cells in both clathrin- and caveolin-mediated manners (McCarthy et al. 2014). The spatial arrangement of the hydrophilic arginines and hydrophobic leucines in the helical structure, enabled by the 12 alanines, confer amphipathicity (McCarthy et al. 2014). It has also been shown that there is an increase in the α-helicity of RALA with a drop in pH from 7.4 to 5.5 which negates entrapment in the endosome and ensures delivery of the cargo into the cytoplasm in the presence of an acidic environment (McCarthy et al. 2014). The ratio of hydrophilic and hydrophobic residues within RALA is critical for functionality. For example, replacement of the hydrophobic residues with hydrophilic rendered the resultant peptide (RGSG) incapable of transfection (Udhayakumar et al. 2017). To date, RALA has successfully delivered plasmids encoding reporter genes (McCarthy et al. 2014), mRNA (Udhayakumar et al. 2017), siRNA (Bennett et al. 2015), DNA vaccines (Rajendrakumar et al. 2017), mRNA vaccines (Udhayakumar et al. 2017) small molecules such as bisphosphonates (Massey et al. 2016) and calcium-based bone substitutes (Huerta et al. 2008) demonstrating broad utility.

The genetic origins of cancer render suitability for gene therapeutics such as small interfering RNAs, micro RNAs, and CRISPR gene editing tools (Rajendrakumar et al. 2017). Indeed, previous research has reported abrogation of the growth of ZR-75-1 breast cancer xenografts when treated with RALA/pFKBPL (Bennett et al. 2015), and the potency of RALA-delivered inducible nitric oxide synthase (iNOS) in a model of breast cancer metastasis. Survival of mice bearing aggressive MDA-MB-231 micrometastases was significantly increased by treatment with nanoparticles comprising RALA and a constitutively active or a transcriptionally regulated iNOS plasmid (McCrudden et al. 2017).

Nitric oxide (·NO) is a gasotransmitter that functions in a range of physiological processes, most notable being the regulation of vascular tone (Huerta et al. 2008). The functionality of ·NO in cancer is concentration-dependent, with low physiological concentrations (in the nanomolar range) provoking a pathological phenotype, and superphysiological concentrations (micromolar) promoting anti-cancer effects (Huerta et al. 2008). It has been postulated that iNOS has a role in the pathogenesis of prostate cancer (Klotz et al. 1998), with iNOS expression levels proportional to Gleason grade, and indicators of proliferation (Ki-67, mitotic index and S-phase fraction) in prostate cancer patients (Aaltoma et al. 2001). However, NOS enzyme [of which there are three isoforms—endothelial (e)NOS, neuronal (n)NOS and iNOS] expression levels do not necessarily equate to ·NO levels, as other enzymes, including the arginases (Heller 2008) and the NOS enzymes’ co-factors BH2 and BH4 (Rabender et al. 2015) also influence ·NO levels. It has been proposed that insufficient iNOS levels may actually enable malignancy, by denying hyperproliferative tissues the therapeutic benefit afforded by ·NO (Heller 2008).

There is a significant body of evidence supporting the use of iNOS as a therapeutic transgene in cancer (Hatefi and Canine 2009; Tambe et al. 2017; Xu et al. 2017; Baltaci et al. 2001; Lee et al. 2009; Reschner et al. 2009; Gannon et al. 2010; Siemens et al. 2009; Holland et al. 2013). The purpose of this study was to investigate whether systemic delivery of the RALA/iNOS nanomedicine was therapeutic in a metastatic model of prostate cancer.

## Methods

### Materials

Unless otherwise stated, reagents used were from Sigma (Dorset, UK).

### Cell culture

PC-3 prostate cancer cells were purchased from ATCC, and maintained in RPMI-1640 (Life Technologies) supplemented with 10% fetal bovine serum (PAA). PC-3M-luc-2 were purchased from Caliper Life Sciences (Buckinghamshire, UK) and maintained in RPMI-1640 (Life Technologies) supplemented with 10% fetal bovine serum (PAA). Cells were cultivated in 175 cm^2^ flasks in a humidified incubator; once 80–90% confluency was reached, cells were passed to maintain exponential growth. Mycoplasma absence was confirmed monthly, using Plasmotest (Invivogen, France).

### Plasmid DNA preparation

MAX Efficiency^®^ DH5α™ Competent Cells transformed with pEGFP-N1 or CMV-iNOS plasmids were cultured in a shaking incubator overnight at 37 °C in Luria broth containing 50 μg/ml ampicillin. Plasmid DNA was isolated and purified using PureLink^®^ HiPure Plasmid Maxiprep Kits (Life Technologies, Paisley, UK), as recommended by the manufacturer. Plasmid DNA, dissolved in ultrapure water, was stored at − 20 °C.

### Nanoparticle complexation and characterization

RALA, supplied as a desalted lyophilized powder was reconstituted in ultrapure water to a stock concentration of 5.8 mg/ml. Aliquots were stored at − 20 °C until use.

Plasmid DNA (pDNA)/RALA nanocomplexes were prepared as described previously (McCarthy et al. 2014); electrostatic interaction between cationic RALA and anionic pDNA (30 min at room temperature) facilitates the formation of particles with size and charge characteristics suitable for gene delivery (McCarthy et al. 2014; Bennett et al. 2015; McCaffrey et al. 2016). Nanoparticles were complexed at *N*:*P*10 (the *N*:*P* ratio is the molar ratio of positively charged nitrogen atoms in the peptide to negatively charged phosphates in the pDNA backbone—at *N*:*P*10, 14.5 μg of RALA is complexed with 1 μg of DNA); nanoparticle size and charge can be altered by modifying the *N*:*P* ratio. For analysis of intracellular nanoparticle behavior, nanoparticles were complexed with RALA conjugated to fluorescein isothiocyanate (FITC) (Biomatik) and pDNA labeled with Cy3 using a Mirus Bio LabelIt^®^ kit (Cambridge Bioscience, Cambridge, UK)). Nanoparticle physicochemical properties were analyzed using a Nano ZS Zetasizer and DTS software (Malvern Instruments, UK).

### Transmission electron microscopy

RALA/DNA complexes were prepared at *N*:*P* 10 with 1 µg pCMV-iNOS in a total volume of 30 µl. Nanoparticles were loaded onto a carbon-coated copper 400 mesh grid (TAAB Laboratories, UK) and allowed to dry. Following drying, the samples were stained with 5% uranyl acetate in methanol at room temperature for 1 min, washed with 50% ethanol then molecular grade water and allowed to dry again. Nanoparticles were imaged using a JEM-1400Plus Transmission Electron Microscope (Joel, USA) at an accelerating voltage of 120 kV. Settings were as follows: pinhole (m) 95.5 µm, pinhole (airy) 999.4 µm, laser (Argon, visible) On (29%), Laser (DPSS 561, visible) On, laser (HeNe 633, visible) On, optical magnification with 10× and a 63× oil immersion objective, whole section depth was 19.13 µm, with 39 *Z* sections. Each section was 0.5 µm thick. The *Z* position of the XY image was 24, meaning 12 µm from the top of the cell.

### Cellular uptake of FITC-RALA/Cy3-pDNA NPs

PC-3M-luc2 were seeded in 24-well plates at 10^4^ cells per well, and incubated overnight. Cells were conditioned for 2 h in Opti-MEM (Life Technologies) before addition of nanoparticle complexes (NPs complexed at *N*:*P* 10), and cells were transfected with NPs equivalent to 0.5 µg DNA per well. Cellular FITC/Cy3 content was assessed over the following 120 h by flow cytometry using a CytoFLEX instrument (Beckman Coulter, Labplan, Dublin, Ireland). FITC and Cy3 contents were assessed using manufacturer settings for FITC and PE-A. For the 120-h timepoint, cells were transferred from the wells of 24-well plates to those of 6-well plates to allow for proliferation.

### Intracellular nanoparticle tracking

PC-3s were seeded in 24-well plates on round coverslips at 10^4^ cells per coverslip, and incubated overnight. Cells were conditioned for 2 h in Opti-MEM (Life Technologies) before addition of nanoparticle complexes, and cells were transfected for 240 min. Cells were fixed using 4% paraformaldehyde in PBS, and coverslips were mounted onto microscope slides using Diamond Antifade with DAPI (Life Technologies). Nanoparticle localization was analyzed by confocal fluorescence microscopy using a Leica SP5 microscope and LAS-AF software.

### Clonogenic assay

PC-3M-luc2 were seeded in T25 culture flasks at a density of 10^6^ cells per flask, and incubated overnight. Following a 2-h starvation in Opti-MEM, cells were transfected with RALA/CMV-iNOS nanoparticle formulations, equivalent to 6 μg DNA per flask; following a 6-h transfection, transfection media were replaced with normal growth medium, and cells were incubated overnight. Following 24 h, cells were trypsinized, counted and plated in triplicate in 6-well plates at 500/1000 cells per well. Plates were incubated at 37 °C for 12 days, following which, colonies were fixed and stained using 0.4% crystal violet (Sigma) in 70% methanol; excess stain was removed by gentle washing in water, and once dry, colonies were manually counted.

### In vivo immunological response to RALA/pDNA nanoparticles

In order to determine whether nanoparticles complexed of RALA and pDNA provoked an immune response, a range of ex vivo assays in C57BL/6 mice were performed. Mice were subjected to single or repeated administrations of PBS, RALA/pEGFP-N1 nanoparticles or polyethylenimine (PEI)/pEGFP-N1 nanoparticles. Each injection delivered nanoparticles (at *N*:*P* 10) equivalent to 10 µg pDNA, and injections were weekly for 3 weeks. 48 h after each injection, three mice were sacrificed; blood was collected by cardiac puncture, and the serum was extracted for analysis of total IgG, IgM, IL-1β, and IL-6 using assay kits (Enzo Life Sciences, Exeter, UK). The significance of the impact on these mediators was assessed using two-way ANOVA with Dunnett’s multiple comparisons test.

To determine whether repeated administrations of nanoparticles provoke neutralizing antibody responses, similar administrations were performed. Following sacrifice, blood was collected by cardiac puncture, serum was isolated, and sera from triplicate mice were pooled, heat inactivated and stored at − 20 °C. 5 × 10^3^ PC-3 were seeded in triplicate wells of 96-well plates and allowed to adhere overnight. Cells were starved in Opti-MEM for 2 h prior to transfection. Freshly prepared RALA/pEGFP-N1 nanoparticles were incubated for 30 min in sera from mice that had received one of the indicated treatments. Sera/nanoparticle mixtures were diluted in Opti-MEM, and used to transfect PC3s. Transfections were for 6 h, following which, Opti-MEM was replaced with RPMI-1640. After 48 h, cells were analyzed for eGFP expression by flow cytometry using a BD FACSCalibur.

### iNOS transgene expression

PC-3M-luc2 were plated (10^4^ cells per well of a 24-well plate) and allowed to adhere overnight, and were transfected with RALA/CMV-iNOS for 6 h, following which Opti-MEM was replaced with phenol red-free MEM/10% fetal bovine serum. (RPMI-1640 is nitrite-rich, which would interfere with the nitrite content assay.) Medium nitrite content was assayed 48 h later using Greiss test for nitrites (Active Motif, Belgium), following the manufacturer’s instructions. Cellular iNOS expression was measured by western blot as previously described (Ning et al. 2012).

### Establishment of metastatic disease

All animal experiments were carried out in accordance with the Animal (Scientific Procedures) Act 1986 and conformed to the current UKCCCR guidelines. Mice were bred in-house and maintained using the highest possible standard of care, and priority was given to their welfare.

Mice (6–8 weeks old) were anesthetized using isoflurane (3% in O_2_) and restrained using surgical adhesive tape in a supine position. Thoracic fur was removed using Veet hair removal cream. Using a 1-ml syringe/26G needle, mice were inoculated with 10^5^ PC-3M-luc2 in 100 μl PBS via the left cardiac ventricle (Lim et al. 2011)—the cell suspension was gently injected into the ventricle, following which the needle was held in place for 10 s to minimize leakage from the ventricle. Mice were imaged using an IVIS200 (Xenogen) instrument to confirm appropriate ventricular delivery. Mice were injected intraperitoneally with 150 mg/kg d-luciferin; following a 15-min incubation, mice were anesthetized using isoflurane and imaged. Appropriate left ventricular delivery is characterized by luminescence throughout the body, while inappropriate delivery is characterized by luminescence that is limited to the thoracic cavity.

### iNOS gene therapy

Gene therapy treatment began 48-h post-inoculation, with mice receiving treatments twice weekly, totaling five treatments. RALA/CMV-iNOS nanocomplexes (corresponding to 5 × 10 μg DNA per mouse) at *N*:*P* 10 were delivered via the tail vein. Solvent (PBS) and vehicle (RALA equivalent to the mass of RALA used in the gene therapy regimen) controls were also performed.

Mice were monitored for micrometastases development using routine IVIS imaging, as well as body mass measurement. A loss of 20% original body mass was deemed sufficient to necessitate sacrifice of the mouse. The degree of whole body luminescence in mice was determined using Living Image software (Perkin Elmer).

### Statistics

All statistics were performed using GraphPad Prism, version 6.0 g for Mac OS X. The tests used are described throughout.

## Results

### Physicochemical characterization of RALA/pDNA NPs

**Fig. 1:**
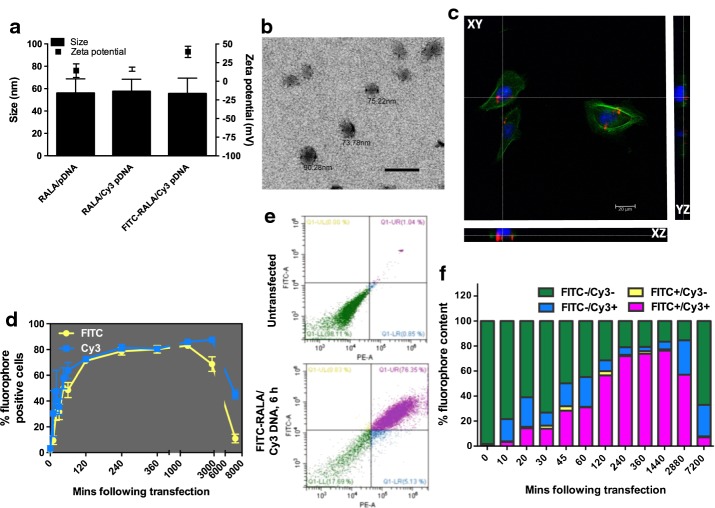
RALA/pDNA nanoparticles are suitable for rapid uptake by PC3 prostate cancer cells. **a** Nanoparticles comprising RALA and pEGFP-1 plasmid DNA have size and charge properties suitable for cellular entry. Conjugation of Cy3 to pDNA does not impact size or charge of nanoparticles, while nanoparticles comprising FITC-conjugated RALA and CY3-conjugated pDNA are of a similar size, but more positively charged. **b** TEM of RALA/CMV-iNOS nanoparticles at *N*:*P* 10; scale bar 200 µm. **c** FITC-RALA/Cy3-pDNA nanoparticles are rapidly taken up by PC3 prostate cancer cells. **d** Orthogonal sectioning of PC3 cells after 4-h transfection with RALA/Cy3-CMV-iNOS nanoparticles. **e** Representative dot plots of FITC/Cy3 positivity in PC3s transfected with FITC-RALA/Cy3-pDNA nanoparticles. **f** PC3s transfected with FITC-RALA/Cy3-pDNA nanoparticles rapidly accumulate and retain both fluorophores up to 1440 min following transfection; FITC content is lost more rapidly than Cy3, with 25% of cells Cy3-positive 7200-min (5 days) post-transfection. Datapoints represent mean ± SEM; *N* = 3

Incubation of plasmid DNA with RALA provoked spontaneous complexation of the two components into cationic nanoscale particles. Labeling pDNA with Cy3 before complexing NPs did not significantly impact the physicochemical properties of NPs (Fig. 1a). Use of FITC-conjugated RALA to condense Cy3-labeled pDNA into NPs afforded the NPs a higher zeta potential than those formed using unlabeled peptide/DNA, although the NP size was unaffected (Fig. 1a). Figure 1b shows a TEM micrograph of RALA/CMV-iNOS nanoparticles at *N*:*P* 10; scale bar represents 200 µm. The apparent diameters of nanoparticles in the TEM image appear to support the diameter data produced by dynamic light scattering. Figure 1c shows the uptake of RALA/Cy3-DNA nanoparticles 4-h post-transfection. Orthogonal sectioning was used to determine the cellular localization of fluorescent DNA; at 4 h, DNA was strongly associated with the cell, and could be seen to be crossing the cell membrane. PC3M-luc2 cells also rapidly took up nanoparticles labeled with both fluorophores (FITC-RALA/Cy3-pDNA—Fig. 1d–f). The profile of cell fluorophore contents progressed in parallel for the first 24 h (1440 min); at 48 h (2880 min), and more pronounced at 120 h (7200 min), Cy3 content was appreciably higher than that of FITC, indicating that the RALA vehicle is discarded, but the pDNA is retained. By 120 h, the Cy3 signal is probably diluted by mitosis, with daughter cells not inheriting the Cy3 label (Fig. 1d, f). Figure 1e contains representative dotplots of FITC/Cy3 content in untransfected and transfected cells with FITC-RALA/Cy3-pDNA (360 min), while Fig. 1f summarizes the pattern of fluorophore content in transfected cells at all timepoints.

### Gene expression following RALA/pDNA transfection

**Fig. 2:**
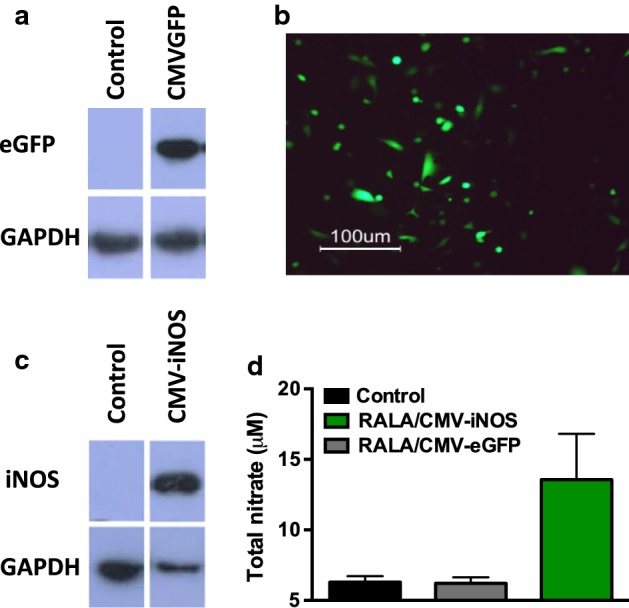
Validation of transgene expression in PC3s. **a**, **b** PC3s were transfected with RALA/pEGFP-N1 (comprising 0.5 μg DNA) at *N*:*P*10 for 6 h. Cells were analyzed for GFP expression 48-h post-transfection using immunoblotting, and fluorescence microscopy. **c**, **d** PC3s were transfected with RALA/CMV-iNOS (comprising 0.5 μg DNA) at *N*:*P*10 for 6 h. Cells were analyzed for iNOS expression 48-h post-transfection using immunoblotting, for ·NO generation by Greiss test. Datapoints represent mean ± SD; *N* = 3

PC3 M-luc2 cells transfected with RALA/pEGFP-N1 displayed eGFP expression 48-h post-transfection (Fig. 2a, b). When PC3 M-luc2 were transfected with RALA/CMV-iNOS, protein was detected 48-h post-transfection (Fig. 2c), and medium nitrate content was 2.2-fold higher than that seen in control conditions; RALA/pEGFP-N1 transfection did not affect extracellular nitrate levels (Fig. 2d).

### RALA/CMV-iNOS treatment inhibits the clonogenicity of PC3M-luc2

**Fig. 3:**
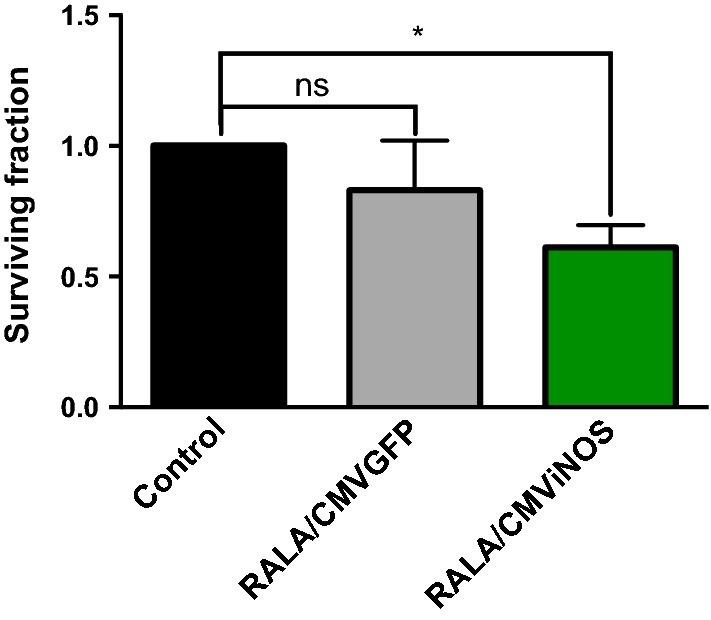
Validation of transgene expression in PC3s. PC3-luc2M cells overexpressing iNOS form fewer clonogenic colonies than control. Datapoints represent mean ± SEM; *N* = 3

PC3M-luc2 cells transfected with RALA/CMV-iNOS had a significant reduction (61.1 ± 8.5%) in clonogenic colonies compared to untransfected cells (100%). Transfection of PC3M-luc2 cells with RALA/pEGFP-N1 (83.0 ± 19.1%) did not affect clonogenicity (Fig. 3).

### Treatment of C57BL/6 mice with RALA/pEGFP-N1 NPs does not affect circulating IgG, IgM, or IL-1β and IL-6 levels

**Fig. 4:**
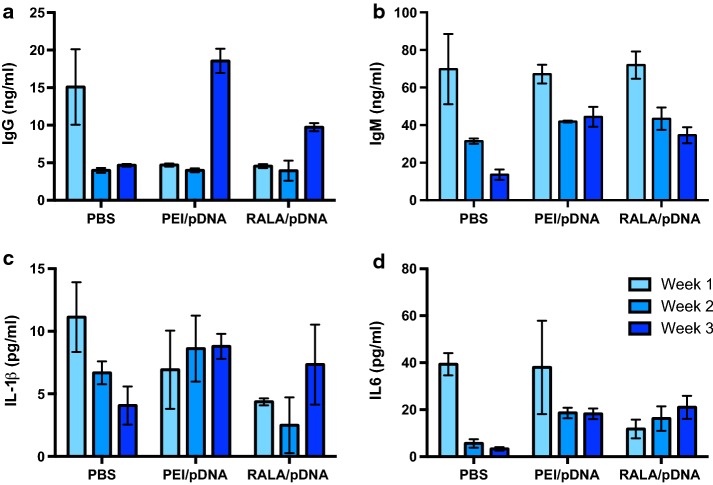
Immune response of C57BL/6 mice injected with PBS, PEI/pDNA, or RALA/pDNA nanoparticles (10 µg pDNA at *N*:*P*10). Mice received one injection per week for 3 weeks. 48 h after each injection three mice were sacrificed and the serum was assayed for IgG (**a**), IgM (**b**), IL-1β (**c**), and IL-6 (**d**) content. Datapoints represent mean ± SEM; *N* = 3

The impact of RALA/pDNA or PEI/pDNA compared to PBS treatment on circulating IgG, IgM, IL-1β, and IL-6 levels are summarized in Fig. 4a–d, and in Table 1.

**Table 1:** Comparisons of treatment compared to PBS only on circulating IgG, IgM, IL-1β and IL-6 levels

Timepoint	Comparison	Mean difference	95% CI of diff.	Significance
IgG				
Week 1	PBS v PEI/pDNA	10.40	4.20 to 16.61	**
	PBS v RALA/pDNA	10.54	4.33 to 16.75	**
Week 2	PBS v PEI/pDNA	− 0.02	− 6.23 to 6.20	ns
	PBS v RALA/pDNA	0.03	− 6.18 to 6.24	ns
Week 3	PBS v PEI/pDNA	− 13.89	− 20.1 to − 7.68	***
	PBS v RALA/pDNA	− 5.07	− 11.28 to 1.15	ns
IgM				
Week 1	PBS v PEI/pDNA	2.66	− 23.05 to 28.37	ns
	PBS v RALA/pDNA	− 2.18	− 27.89 to 23.52	ns
Week 2	PBS v PEI/pDNA	− 10.44	− 36.15 to 15.27	ns
	PBS v RALA/pDNA	− 11.99	− 37.7 to 13.72	ns
Week 3	PBS v PEI/pDNA	− 30.79	− 56.5 to − 5.08	*
	PBS v RALA/pDNA	− 20.98	− 46.69 to 4.73	ns
Il-1β				
Week 1	PBS v PEI/pDNA	4.20	− 3.29 to 11.69	ns
	PBS v RALA/pDNA	6.77	− 0.72 to 14.26	ns
Week 2	PBS v PEI/pDNA	− 1.94	− 9.43 to 5.55	ns
	PBS v RALA/pDNA	4.19	− 3.31 to 11.68	ns
Week 3	PBS v PEI/pDNA	− 4.73	− 12.22 to 2.76	ns
	PBS v RALA/pDNA	− 3.27	− 10.76 to 4.22	ns
IL6				
Week 1	PBS v PEI/pDNA	− 27.61	− 52.87 to − 2.35	*
	PBS v RALA/pDNA	− 26.24	− 51.50 to 0.97	*
Week 2	PBS v PEI/pDNA	10.61	− 14.66 to 35.87	ns
	PBS v RALA/pDNA	− 2.41	− 27.67 to 22.85	ns
Week 3	PBS v PEI/pDNA	17.74	− 7.52 to 43.00	ns
	PBS v RALA/pDNA	2.80	− 22.47 to 28.06	ns

*p < 0.05; **p < 0.01; ***p < 0.001

### RALA/pEGFP-N1 NPs provoke no neutralizing antibody response in immunocompetent mice

**Fig. 5:**
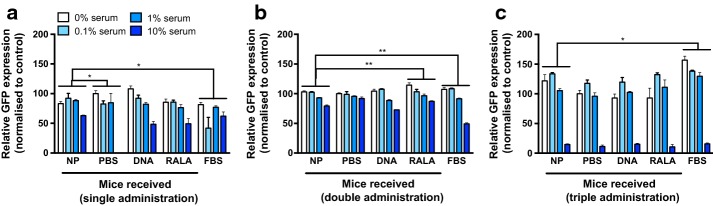
Administration of RALA/pEGFP-N1 nanoparticles to immunocompetent mice does not provoke a neutralizing antibody response. Flow cytometric analysis of GFP in PC3s after incubation of RALA/pEGFP-N1 nanoparticles with sera from C57BL/6 mice that received the indicated treatment (PBS/DNA/RALA/NPs) weekly for up to 3 weeks (**a** Week 1; **b**. Week 2; **c**. Week 3). *p < 0.05, **p < 0.01 compared to expression elicited by RALA/pEGFP-N1 nanoparticles (NP) that had been incubated in sera from mice that had received nanoparticles (multiple comparisons ANOVA)

Incubation of RALA/pEGFP-N1 NPs with sera from C57BL/6 mice previously treated with RALA/pEGFP-N1 did not appreciably affect transfection ability of the NPs. One-way ANOVA with Dunnett’s correction for multiple comparisons was used to compare sera from nanoparticle-treated mice with other treatments. In no case did incubation in sera from nanoparticle-treated mice lessen eGFP expression (Fig. 5a–c). Increasing serum concentration slightly abrogated the transfection ability of NPs, although the relevance of this is questionable. Any inhibition of transfection ability cannot be attributed to NP neutralization, as this abrogation was observed in all sera, including FBS, which had not been pre-exposed to nanoparticles. The ‘significance’ of transfection inhibition between mice that received nanoparticles and those that received PBS at week 1 can likely be explained by the absence of a 10% serum datapoint in the PBS group.

### RALA/CMV-iNOS therapy delays the progression of metastatic prostate cancer

**Fig. 6:**
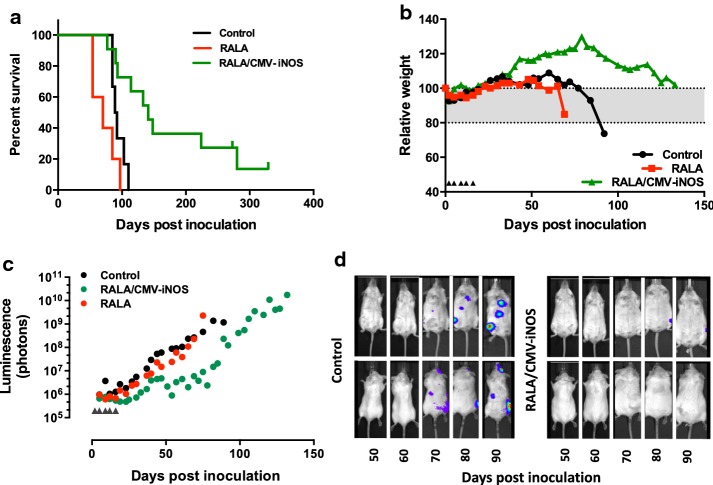
Treatment with RALA/CMV-iNOS improves survival of PC3-luc2M metastases-bearing mice. **a** Survival of metastases-bearing mice. *N* ≥ 5 (control, RALA) or 11 (RALA/CMV-iNOS). **b** Weight loss of exemplar mice. **c** Total bioluminescence in exemplar mice; inverted triangles denote treatment timepoints. **d** IVIS images of mice (control and RALA/CMV-iNOS) showing bioluminescence accumulation at indicated timepoints post-implantation

Treatment of mice bearing metastatic foci of PC3M-luc2 with RALA/CMV-iNOS significantly increased median survival from 90.5 days (control) to 141 days (*p* = 0.005). RALA alone did not significantly alter median survival (*p* = 0.0824) (Fig. 6a). Mice that received RALA/CMV-iNOS treatment lost weight and developed bioluminescence more slowly than control or RALA-treated counterparts. Figure 6b represents the degree of relative weight loss of individual mice whose post-inoculation survival was closest to the relevant treatment’s median value; Fig. 6c represents bioluminescence accumulated in the same mice. For cumulative weight loss and bioluminescence accumulation see Supplementary Figs. 1 and 2. 50% of the mice in the RALA/CMV-iNOS treatment group were sacrificed without having lost 20% original body weight. Similarly, these mice also progressed though the study developing considerably less bioluminescence than others in the same treatment group. In that respect, these mice could be considered to be complete responders, being devoid of the two indicators of disease that were studied, namely weight loss and bioluminescence. Figure 6d displays the evolution of metastatic foci in the individual control and RALA/CMV-iNOS-treated mice whose physical and biochemical data are presented in Fig. 6b, c.

## Discussion

It was previously reported that RALA/iNOS nanoparticulate gene therapy produced ·NO production in vitro, and improved survival following systemic administration in a model of breast cancer metastasis (McCrudden et al. 2017). The results reported here reinforce those findings in prostate cancer. The improvement in survival in this prostate cancer model was much higher (56% increase in median survival in the PC-3 model versus a 27% increase in median survival in the breast cancer model), although an explanation for this has not been ascertained. MDA-MB-231 breast cancer cells are a notoriously aggressive model of breast cancer, and the progression of disease was faster in that model (the median survival of control mice in this study was 90.5 days, but only 31.5 days in the breast model), which may account for the observed differences (McCrudden et al. 2017).

Therapeutic ·NO is most commonly achieved using a donor drug such as organic nitrates, metal-NO complexes, *S*-nitrosothiols, sydnonimines, diazeniumdiolates (NONOates), and ·NO-drug hybrids (Huerta et al. 2008). In contrast, our strategy of using a simple peptide and native DNA as a therapeutic should provoke fewer drug-related side effects than these donor agents or viral vectors would (Hatefi and Canine 2009). RALA/CMV-iNOS nanoparticles were in the same size range as nanoparticles used by others in prostate cancer nucleic acid therapies (Tambe et al. 2017; Xu et al. 2017). Nanoparticles delivered intravenously are readily taken up by organs such as the lungs, liver, and spleen, so these organs are particularly susceptible to possible off-target side effects. Expression of iNOS was not assessed in these organs although it has been previously shown that the lungs and liver strongly express transgenes following RALA/DNA delivery via the tail vein (McCarthy et al. 2014). Although no harmful side effects were observed, strategies to prevent iNOS expression in non-target tissues, such as transcriptional targeting (McCrudden et al. 2017), are still an attractive proposition.

iNOS and ·NO may be controversial agents to employ for the treatment of cancer. Correlations between iNOS and prostate cancer have been described previously (Klotz et al. 1998). Indeed, high-grade prostatic intraepithelial neoplasia (PIN) and prostatic carcinoma samples had higher iNOS expression than low-grade PIN or benign prostatic hyperplasia specimens (Baltaci et al. 2001). Polymorphisms in the iNOS gene were associated with prostate cancer aggressiveness, although the functional consequences of these polymorphisms are unclear (Lee et al. 2009). However, despite these correlations, an argument exists that cancers develop as a result of failure to achieve sustained high-level (and therefore therapeutic) ·NO production, rather than being consequent of iNOS expression. Arginase, which depletes iNOS’s substrate, l-arginine (Heller 2008), is overexpressed in prostate cancer tissue (Reschner et al. 2009). As arginases may interfere with the therapeutic activity of iNOS, it is possible that selective inhibition of arginase activity could further boost the potency of therapeutic strategies that aim to achieve elevated ·NO levels, such as our RALA/iNOS therapy. The first trial assessing the safety and tolerability of a selective arginase inhibitor, CB-1158, is currently recruiting patients with solid tumors (NCT02903914). Indeed, the therapeutic benefit of androgen deprivation therapy in patients may be in-part down to inhibition of the expressions of both arginases 1 and − 2 (Gannon et al. 2010). Other mediators that affect iNOS, such as *N*-chlorotaurine or *N*-bromotaurine, have been shown to impact upon the concentration and (patho)physiology of intratumoral ·NO (Heller 2008).

A concern associated with the delivery of ·NO is the possibility that vasodilation may manifest as hypotension. The present study did not find any evidence of toxicity of RALA/CMV-iNOS therapy consistent with hypotension, nor did our previous study of RALA/iNOS strategies in breast cancer (McCrudden et al. 2017). Non-localized delivery has been observed to produce impressive therapeutic efficacy in the past—a nitroglycerin-releasing transdermal patch almost trebled the PSA doubling time in a cohort of prostate cancer patients post-surgery or—radiotherapy (Siemens et al. 2009). Notwithstanding that, a strategy to limit ·NO release to the target tissue to preclude systemic toxicities is attractive. Several ·NO donor drugs have been designed to include strategies to ensure ·NO production is limited to the tumor, including β-galactosidase-provoked release of ·NO/HNO from IPA/NO (Holland et al. 2013), nitroreductase-dependent ·NO release from 1-(2-methylpiperidin-1-yl)diazen-1-ium-1,2-diolate (Sharma et al. 2013), and the preferential release of ·NO from RRx-001 in hypoxia (Ning et al. 2012). The distinctive tumor phenotype can be exploited for targeting genetic therapies also; functionalization of nanoparticles with anti-prostate-specific membrane antigen (PSMA) RNA aptamer facilitated precise miRNA delivery to xenografts following systemic administration, and impressive tumor growth delay (Binzel et al. 2016). Xu and colleagues used a pH-responsive polymer to provoke nanoparticle disassembly and siRNA delivery in the acidic tumor microenvironment, and reported marked tumor growth delay in an LNCaP xenograft model of prostate cancer (Xu et al. 2017).

The use of the human osteocalcin promoter to facilitate transcriptional targeting, thereby limiting iNOS transgene expression to tumors with elevated RUNX2 expression has also been employed (McCrudden et al. 2017). Future studies will focus on the functionalization of RALA/DNA nanoparticles to facilitate homing to tumors. The benefits of vehicle modification were demonstrated by Lee and colleagues, who through nanoparticle modification (thiol-modified glycol chitosan), produced a nucleic acid-delivery vehicle that bypassed normal tissue, preferentially delivering to PC-3 xenografts. The unembellished nanoparticles had modest potency, but replacement of the chitosan with the thiolated chitosan markedly improved the anti-tumor benefit of systemically delivered siVEGF (Lee et al. 2017).

## Future directions and conclusions

For further evaluation of the RALA platform, a more comparative study could be performed with respect to other liposomal or polymeric delivery systems to not only compare efficacy but also toxicity both in vitro and in vivo. Additionally, a wider panel of cytokine markers could be measured in vivo to support the initial findings of no significant inflammatory response. To develop this therapy further, variables such a dose, timing and duration required optimization. Despite no adverse toxicity with constitutively drive iNOS, targeted delivery could be considered. This could be achieve via transcriptional targeting of the gene (McBride et al. 2016) or by amending the RALA platform to target tumors using ligands such as TMTP-1 (Coulter et al. 2010). Nevertheless, the data presented in this study demonstrate that the DNA persists in the cell after the RALA is disassociated, that RALA does not produce any neutralizing antibodies and that IgG and IgM effects are negligible following repeated injection. There is also a clear anti-cancer effect of RALA/iNOS gene therapy for metastatic prostate cancer following both in vitro and in vivo studies. Although the mechanism by which ·NO achieves this anti-tumor effect in PC3 metastases has not yet been determined, previous studies have indicated that the production of dinitrogen trioxide is responsible for toxicity (Ali et al. 2013), inhibition of angiogenesis or recruitment of cytotoxic T cells (Singh and Gupta 2011). This research supports the continued development of iNOS gene therapy in the non-viral RALA delivery platform.
